# Orientation in high-flying migrant insects in relation to flows: mechanisms and strategies

**DOI:** 10.1098/rstb.2015.0392

**Published:** 2016-09-26

**Authors:** Andy M. Reynolds, Don R. Reynolds, Sanjay P. Sane, Gao Hu, Jason W. Chapman

**Affiliations:** 1 Computational and Systems Biology Department, Rothamsted Research, Harpenden, Hertfordshire AL5 2JQ, UK; 2 Natural Resources Institute, University of Greenwich, Chatham, Kent ME4 4TB, UK; 3 Department of Agroecology, Rothamsted Research, Harpenden, Hertfordshire AL5 2JQ, UK; 4 National Centre for Biological Sciences, Tata Institute of Fundamental Research, Bangalore 560 065, Karnataka, India; 5 College of Plant Protection, Nanjing Agricultural University, Nanjing, People's Republic of China; 6 Centre for Ecology and Conservation, University of Exeter, Penryn, Cornwall TR10 9EZ, UK; 7 Environment and Sustainability Institute, University of Exeter, Penryn, Cornwall TR10 9EZ, UK

**Keywords:** flight orientation, flow sensing, optomotor responses, turbulence directionality cues, migration strategies

## Abstract

High-flying insect migrants have been shown to display sophisticated flight orientations that can, for example, maximize distance travelled by exploiting tailwinds, and reduce drift from seasonally optimal directions. Here, we provide a comprehensive overview of the theoretical and empirical evidence for the mechanisms underlying the selection and maintenance of the observed flight headings, and the detection of wind direction and speed, for insects flying hundreds of metres above the ground. Different mechanisms may be used—visual perception of the apparent ground movement or mechanosensory cues maintained by intrinsic features of the wind—depending on circumstances (e.g. day or night migrations). In addition to putative turbulence-induced velocity, acceleration and temperature cues, we present a new mathematical analysis which shows that ‘jerks’ (the time-derivative of accelerations) can provide indicators of wind direction at altitude. The adaptive benefits of the different orientation strategies are briefly discussed, and we place these new findings for insects within a wider context by comparisons with the latest research on other flying and swimming organisms.

This article is part of the themed issue ‘Moving in a moving medium: new perspectives on flight’.

## 1. Introduction

The sampling of insects migrating high in the air started as early as the 1920s and 1930s [1], but the realization that these insects could exhibit sophisticated ‘in-flight’ behaviour had to wait until the application of radar to entomology in the late 1960s [2,3]. It then became evident that nocturnal migrants, cruising at altitudes of several hundred metres above the ground, frequently shared well-defined heading directions that might persist for an hour or so and, in the larger species at least, cause an individual's trajectory to differ significantly from that of the wind ([3], and references therein). Frequency distributions of headings recorded over a short period (approx. tens of minutes) could be remarkably tight with circular standard deviations (*s*) approximately 15° (e.g. [4]) although *s* values of approximately 30° might be more typical. An *individual's* alignment is also stable over short timescales (a few seconds)—there is no evidence of rapid yawing around the mean direction [3, p. 246].

The migrants are generally too far apart for orientation to be maintained by visual reference to each other [5] and, in any case, mutual references of this sort would show signs of drift in mean headings over the very large areas (hundreds or even thousands of square kilometres) over which orientation patterns have been observed [6–9]. This implies that orientation cues are uniformly present over similar areas, and so the phenomenon is not generally a response to local ground features. (There is very little evidence that *high-flying* migrants react to ‘leading lines’ on the ground [3]—there is one report of large migrating insects occasionally changing their flightpaths so that they became channelled along the course of a large river [10], and smallish day-flying insects have been found to react to coastlines in some circumstances [11]). Despite the normally broad-scale nature of the flight orientations, there are some examples of bimodal heading distributions, i.e. where different species take up different orientations in response to the same aerial environment [4,5].

For some time, the function of this ‘common orientation’ phenomenon among high-flying migrants was unclear—did it materially improve a migrant's ability to reach an ecologically appropriate destination, or did it have another function (e.g. to reduce dispersal in a migrating population [12] or improve flight stabilization [3, p. 243])? Recent radar studies have demonstrated, however, that the flight orientations (along with flight-altitude selection) optimize displacement in seasonally favourable directions in some UK Lepidoptera such as *Autographa gamma* (the silver Y moth) [13–16].

The early radar studies established that orientation direction is generally related to wind direction. For example, on some occasions, the mean heading closely followed the downwind direction despite veering of the wind with altitude [2,17]. In other cases, a (relatively large) off-wind orientation angle to the wind was maintained after a substantial shift in the wind direction [4,18]. There was also evidence that insects take account of wind velocity by flying preferentially at altitudes with fast-moving and stable wind streams (see references in [19]). In some of these cases, the insects appear to be reacting to a wind-related feature [19], rather than a proxy cue for wind speed (such as local maxima in air temperature at the top of an inversion). In an interesting, although unusual, group of cases from the Middle Niger area in Mali, West Africa, night-flying insects (probably acridoid grasshoppers) were observed to head towards, and move in, a preferred geographical direction (approx. 30°–40°) in light winds (approx. 2–4 m s^−1^) from varying directions but with a distinct upwind component [4]. Although this indicates that the insects involved were using some sort of compass sense, they must still have perceived that the wind at high altitude was light enough for them to achieve this movement, because backwards drift was not observed.

Despite many informative case studies, investigations with the early scanning radars were constrained by their labour-intensive operation and data-extraction methodologies. Recently, very long runs (approx. 10 years) of data from continuously operating entomological radars have been analysed including, for the first time, extensive records from day-flying migrants [20]. These analyses revealed that, where migrants were numerous enough to form analysable events, wind-related orientations were extremely common, almost ubiquitous, in medium-sized (approx. 10–70 mg) insects flying in the day as well as at night.

The question thus arises as to how the high-altitude wind-mediated headings are selected and maintained and, especially, what sensory modalities are being used by the migrants. Here we review the evidence for the candidate mechanisms, postulate a new ‘turbulent jerks’ mechanism and consider how the various types of observed orientation might form part of an adaptive migration strategy. Finally, we discuss parallels and dissimilarities of the insect orientation cues to those used by other flying and swimming taxa, with special reference to the utility of turbulence in providing cues for different types of organisms, ranging from jellyfish to birds.

## 2. Mechanisms for the selection and maintenance of wind-related orientation

Orientation with reference to the concurrent wind velocity seemingly relies on either visual responses to the apparent movement of ground images over the ommatidia of the insect's eye, and/or is sensed through turbulent velocity or temperature structures in the atmosphere [4,5,19,21,22]. These mechanisms are now discussed in turn.

### (a) Visual perception of relative ground movement

Insects rely heavily on the optomotor response and other visually mediated behaviours for flight stabilization and manoeuvres near the ground (e.g. [23,24]). For example, locusts take off more or less into the wind, and then climb until they are about to be blown backwards. This course is not tolerated and so the insect turns down- or crosswind. However, if an individual continues to climb, and the wind speed increases with height, there will come a point where a ‘preferred retinal velocity’ may be exceeded, which might provoke descent and landing (e.g. [25]). This apparent difficulty was rationalized by postulating a ‘maximum compensatory height’ (m.c.h.) above which the ‘grain size’ of the ground pattern will no longer be optically resolvable and/or the speed of image movement will be too slow to evoke a response [25]. Above the m.c.h., therefore, an optomotor response would no longer operate and the migrant would be free to be carried by the wind (with no wind-related orientation). There are occasional examples where this appears to occur, even in broad daylight, e.g. in the painted lady butterfly, *Vanessa cardui*, migrating up to at least 300 m above the ground where ‘many appeared to be drifting … as though allowing themselves to be carried NW by the wind. Some of these drifters were spinning slowly (like drifting leaves) with no attempt to maintain a constant orientation’ [26].

The ‘maximum compensatory height’ concept would, nonetheless, seem to require modification in view of the regular occurrence of narrow distributions of wind-related headings at high altitude, both during the day and at night. It would appear that, as a migrant insect continues to climb, the optomotor reactions shown near the surface must become untenable or are deliberately overridden. If migrants are indeed monitoring drift by the apparent movement of ground features, they must be replacing a particular ‘grain’ size in the ground pattern with another (coarser) one as they ascend, so that pattern elements continue to be resolvable.

Unless there are considerable wind speed increases with altitude, the higher the insect flies the slower will be the angular velocity of ground features passing beneath it; thus a deterioration in the precision of downwind orientation with altitude would be expected if vision is the primary modality for wind-related orientation. In fact, the contrary seems to be the case—the angular dispersion of headings observed by radar tends to *decrease* with altitude even when there is little change of wind speed with height [5,17,20,21]—a result more in keeping with a turbulence cue (see fig. 1 in [21]) rather than a visual one. However, in some cases, the apparent improvement in orientation performance with altitude may be confounded by changes in species composition, so further investigation of this topic is required.

**Figure 1.**
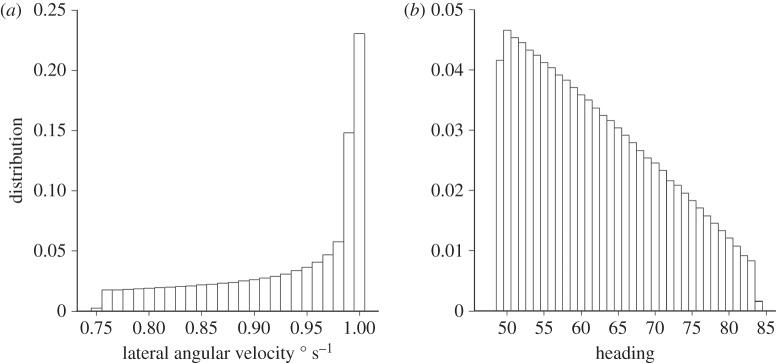
(*a*) Distribution of lateral angular velocities for insects that showed a heading distribution with a range of ±25°, at an angle of 74° to a wind of 10 m s^−1^ at 570 m above the ground (cf. [4]). Lateral angular velocities range between 0.759° s^−1^ (for heading 74°) and 1.005° s^−1^ (for heading 90°) with mean 0.936° s^−1^. (*b*) The skewed distribution of headings that would arise if the insects oriented themselves with a symmetrical distribution of transverse angular rates ranging between 0.759° s^−1^ and 1.005° s^−1^. Headings range between 49.1° and 84.2°, with mean 62.5°.

Although the angular velocities at which ground features appear to move would be very slow at high altitudes, the possibility that insects can sense wind drift to some extent by visual reference to the ground during the day or in moonlight seems, on the face of it, quite plausible. More problematic is whether the mechanism would function at altitudes up to 1 km or more on dark nights, as radar observers agree that common orientation occurs under all illuminance levels [2,4,5]. It should be noted that examples of orientation have been observed in remote locations (e.g. in Mali and Niger) where there were few artificial light sources on the ground that might, potentially, have facilitated visual perception of drift [2,4,17].

Some insects are known to have extremely sensitive night vision [27–29]; for example, the halictid sweat bee, *Megalopta genalis*, can see landmarks well enough to navigate through the understory of a tropical forest at night when illumination levels from the background foliage were as low as 2 × 10^−5^ cd m^−2^ (10–20 times dimmer than starlight illumination) [30]. Moths, in particular, are noted for their ability to see extremely faint visual cues, and possess scotopic colour vision (e.g. [31]). Astonishing as these feats are, insects foraging near the surface are able to use angular rates of background image motion which are much higher than those available to high-flying migrants (and in some cases, the forager may hover and use temporal and spatial photon summation to improve sensitivity in very dim light). As far as insects flying at high altitudes are concerned, a key question might be: can they respond to transverse angular velocities over the ground within the range of about ±0.3–0.1° s^−1^ when terrain luminance was as low as 2 × 10^−8^ lamberts (6.37 × 10^−5^ cd m^−2^) and where the viewing platform (the insect's eye) will presumably be subject to some atmospheric turbulence [32]. The tentative conclusion in that case [32] was that the observed degree of close orientation to the wind did not seem possible in starlight illumination levels, although crude differentiation between, say, approximate crosswind and along-wind flight may have been achievable.

Another issue concerns predictions from optical orientation models which depend on the insect being able to maintain certain angular rates of lateral and/or longitudinal movement in relation to the ground using optomotor anemotaxis (for instance, to orientate with the flow the migrant could adjust its heading so that the apparent ground movement lateral to its body axis tends to zero). Such expectations do not seem to be met under some observed (particularly crosswind orientation) conditions. If, for example, insects were heading at an angle > 45° away from the downwind and showing a symmetrical frequency distribution of headings, then the resulting angular velocity distribution would be highly skewed (figure 1*a*). Consequently, if one postulates that the insects orientate so that the speed of apparent ground movement transverse to their body axes was not zero, but some preferred value, then the expected symmetric distribution of transverse angular rates about a preferred mean would result in a highly skewed heading distribution (figure 1*b*). However, *this is not what is observed*—headings are generally rather symmetrical about the mean (figure 3*c*) and not significantly different from a von Mises (circular normal) distribution [4].

Alternatively, one might postulate that the migrants maintain *a ratio* of lateral to apparent backward angular rates (i.e. a specific drift angle) assuming both rates were high enough to be perceived (cf. [33]). However, this would still not predict the observed symmetrical distributions for off-wind headings (although it would account for the observed *altitude independence* of both the standard deviation and the direction of off-wind headings) [4,5].

### (b) Turbulence-induced cues

The alternative to a visual mechanism is that some intrinsic feature of the wind itself enables insect sensing of wind direction at high altitude. Such ideas have a long history (see references in [4]) but have been given a precise conceptualization only recently. There appear to be three putative mechanisms: (i) temperature ‘ramp-cliff’ structures [22]; (ii) a turbulent velocity mechanism [21,22] and (iii) a new theory (which we propound below) namely, a turbulent ‘jerks’ mechanism.

‘Ramp-cliff’ patterns are observed in a variety of turbulent shear flows in the atmosphere and are characterized by a gradual increase in temperature by as much as several degrees (the ramp) followed by a sharp decrease (the cliff) (or the order may be reversed in ‘cliff–ramp’ patterns). In each case, the cliffs form along the line of diverging flow between eddies. The suggestion was that a migrant aligned with the wind direction would detect near-periodic temperature fluctuations, but would feel more random fluctuations if flying crosswind [22]. There are no size restrictions for this theory, so the cues could, in principle, be used by migratory insects and by birds. The temperature ramps would be expected to show associations with gradients in either mean temperature or mean wind-speed shear, but an analysis of radar data from the UK produced no evidence that the degree of common orientation in nocturnally migrating insects was associated with such gradients [22].

Turning to how migrants might deduce the mean wind direction from turbulent velocity cues, the main concern has been the perception that small-scale eddies in the atmosphere are locally isotropic (i.e. invariant with respect to direction), even if these motions were derived from larger-scale anisotropic motions. Reynolds *et al*. [21,22] formulated fluid-dynamic models suggesting how small-scale turbulent velocity fluctuations and turbulent accelerations might nonetheless provide cues by which insects might align themselves approximately with the direction of the wind flow and, in fact, also account for wind-related layering [19,21,34]. A key prediction from these models was that insects using the wind-mediated cues will be somewhat ‘misled’ by the action of the Ekman spiral – the deflection of the mean wind direction owing to the Coriolis effect so that, in the Northern Hemisphere, surface winds blow to the left of winds aloft (and vice versa in the Southern Hemisphere). When the Ekman spiral is in full effect, insects responding to the turbulence cues should (theoretically) tend to head to the *right* of the mean wind line in the Northern Hemisphere (and to the *left* in the Southern Hemisphere). This prediction is best tested by the orientations of medium-sized insects, which might be expected to adopt a relatively unsophisticated strategy of heading downwind (see §4*b* below). Several extensive studies have now shown that *nocturnal* insects in this size category (body mass 10–70 mg) migrating over the UK routinely exhibit common orientation aligned close to the downwind direction, but typically offset to the right of the flow by an average of approximately 10–20° [20,21,34].

In contrast with a strategy of straightforward downwind orientation, some larger-sized Lepidoptera like *A. gamma* in the UK exhibit a complex strategy of ‘compass-biased downstream orientation’ (CBDO), one element of which involves deviating their heading a certain amount from the flow direction towards a seasonally preferred direction of movement (PDM) in order to (partially) correct for drift [15,35]. These migrants still need to detect the downwind direction, of course, but one would expect that the telltale presence of right offsets indicating the turbulence mechanism might be masked by the above-mentioned shift of headings towards the PDM. However, a careful analysis revealed that turbulence-induced offsets were still visible in the moth drift corrections, because offsets were considerably larger when the wind direction was to the left of the PDM (when turbulence-induced offsets and the drift corrections would both be on the right and thus additive), than when the flow was to the right of the PDM (when the two offsets would oppose each other) [36]. This finding did not apply to nocturnally migrating songbirds, indicating that these do not use turbulence to detect the flow, and presumably rely on visual assessment of drift to infer the flow direction [36].

As the Ekman spiral is typically not present in convective daytime atmospheres, the orientation of day-flying and crepuscular insects would not be expected to show significant directional bias in the flight heading offsets, and this is what was observed (i.e. headings were not systematically offset to either the left or the right of the flow) [20]. The mechanism by which these diurnal and crepuscular migrants identify the flow direction is currently unclear; it could be visual or turbulence-related, or a combination of both [20].

Further evidence in support of the turbulence mechanism could be obtained by observations in the Southern Hemisphere, testing for *left-of-wind-line* offsets, as predicted by the theory. However, the only systematic observations in the Southern Hemisphere were recorded by an insect-monitoring radar in inland New South Wales (Australia), and it was found that night-flying Australian plague locust (*Chortoicetes terminifera*) orientations were related to the wind but were shifted to the right of the downwind when locusts were moving northwards (the predominant situation in the prevailing winds from the east) but orientations were left-shifted when the insects were moving southwards [37]. While these results do not support the turbulence theory, they do not necessarily contravene it either, because strong-flying insects like *C. terminifera* may have complex orientations of the CBDO sort, and these orientations may well obscure any relatively subtle effects of the Coriolis response.

The sensory processes by which airborne insects actually detect small air flows remain to be elucidated. The turbulent velocity mechanisms postulated [21,22] will be quite weak, and so mechanoreceptors on the antennae (particularly those associated with Johnston's organ on the antennal pedicel [38,39]) or wind-sensitive setae on the head and prosternum [40] may have the advantage that they are positioned in front of the vortices produced by the flapping wings and so may be better at detecting small differential pressures on either side of the body. Recent findings show that the Johnston's organs are range-fractionated, i.e. they are capable of encoding antennal vibrations of low to high frequencies with exquisite sensitivity [38,41]. Although such high-frequency sensors may serve as turbulence sensors, the ‘jerks’ mechanism proposed in the next section does not demand sensitive mechanoreceptors specialized for detecting the weak airflows.

## 3. A new turbulence mechanism: anisotropic jerks

Our original theory [21,22] identified a putative mechanism by which migratory insects could determine the mean wind direction from cues provided by turbulent velocity fluctuations. This leaves open the question as to whether or not these turbulence cues would be obscured by disturbances of the surrounding airflow created by wing flapping, and leaves open the identification of the sensory organs. Preliminary numerical simulations of wing flapping suggest that such ‘flight noise’ amplifies rather than masks the turbulent cues (AM Reynolds 2016, unpublished) but these studies have not been verified either theoretically or experimentally. Similarly, some insects have body areas with abundant airflow-detecting mechanosensory hairs, some of extreme sensitivity (e.g. [42]) but these groups of sensors have various functions, and it is not clear if they could operate when migrants are in flight. Here we show that such open questions could be sidestepped, and confidence in the turbulence mechanisms bolstered, by showing that its predictions are not specific to the original modelling assumptions. We show that because migratory insects by virtue of their inertia necessarily lag behind the turbulent air currents, they experience ‘jerks’ (also known as ‘jolts’ and defined as the first derivative of acceleration); these are minimized when the insects are flying downwind, or to the right of the mean wind line when the Ekman spiral is present in the Northern Hemisphere. Jerks are rapidly changing accelerations that tend to produce a loss of flight control (and also whiplash effects), and so will be felt without the need for specialist sensory organs. The mechanism is much more robust in respect of cues being masked by flight noise because the wing-flapping process itself is likely to produce stable flight that *would contrast with the turbulence-induced jerks by the wind*.

### (a) An illustrative example

Consider for illustrative purposes an inert body moving in a one-dimensional turbulent stream. The velocity of the body, *v*, can be related to that of the surrounding air stream, *u*, by Stokes law,
3.1
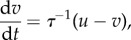
where *τ* is the body's characteristic aerodynamic response time. The accelerations, *A*, and velocities, *u*, of the surrounding air stream can, as shown in the electronic supplementary material, be modelled stochastically by
3.2
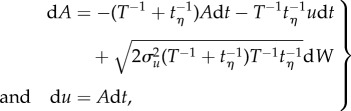
where 
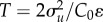
 and 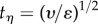 are the two timescales of turbulence, describing the largest scales of motion and the small (dissipative) scales of motion, *σ_u_* is the standard deviation of the turbulent velocity fluctuations, *C*_0_ is Kolmogorov's constant, a universal constant of turbulence, 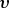 is viscosity and *ɛ* is the rate of change of turbulent kinetic energy, 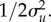 The quantity d*W* is Gaussian noise with mean zero and variance d*t*. Equations (3.1) and (3.2) can be combined into a single set of equations for the jerks, *J*, (and accelerations *A*′) experienced by the body
3.3
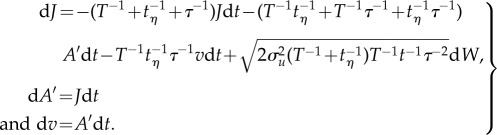


This can be rewritten as
3.4
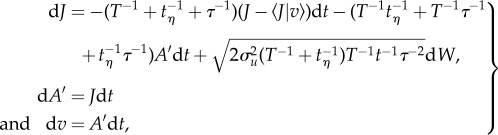
where
3.5
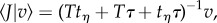
can be recognized as the conditional average jerk. This can be seen by carefully comparing equation (3.4) with equation (3.2), as both equations have the same form. But whereas the first term in equation (3.2) causes accelerations to be centred on zero, the first term in equation (3.4) causes jerks to the centred on 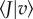, i.e. have conditional average given by 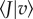. For larger migrants (with 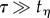), airborne in atmospheric turbulence (which has 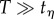), equation (3.5) reduces to 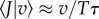 and as a consequence the mean amplitude of the jerks experienced by the migrants is:
3.6
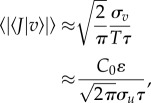
where *σ_v_* is the standard deviation of the velocity of the body, which here is assumed to be approximately equal to *σ_u_*. This assumption is justifiable when the body is responsive to most turbulent velocity fluctuations in the surrounding airstream, i.e. when *τ* < *T*. The mean amplitude of the jerks experienced by the migrants is thus dependent on the turbulent velocity fluctuations of the airstream, i.e. on *σ_u_*. As a consequence, in three-dimensional turbulence, the average magnitudes of the jerks experienced along the body-line will be minimized when the migrant is flying downwind (or to the right of the mean wind line when the Ekman spiral is present), i.e. in the direction in which turbulent velocity fluctuations are largest.

Similarly, for small migrants (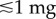) with 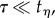 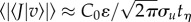 but this orientation cue will be of little value because the jerks will inhibit flight control, and so inhibit the maintenance of their heading. In the original theory, the observed absence of common orientation in small insects was attributed to the absence of orientation cues.

These results are not specific to the modelling assumptions used above and can be deduced from general principles.

### (b) Deriving the result from general principles

The distribution of jerks, accelerations and velocities experienced by a migrant can, in general, be modelled by
3.7
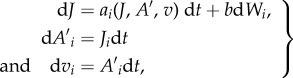
where the subscripts denote Cartesian coordinates. Equation (3.3) is the simplest example of this. The functions and *a* and *b* are determined by the Fokker–Planck equation
3.8
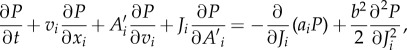
where *P* is the joint distribution of positions, velocity, accelerations and jerks. Equation (3.3) corresponds to the case when these velocities, accelerations and jerks are stationary, homogeneous and Gaussian. Here for simplicity (but without loss of generality), *P* is taken to be both stationary and homogeneous. Integrating equation (3.8) over all jerks and then over all accelerations then gives
3.9a
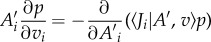
and
3.9b
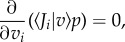
which implies that the conditional average jerks are non-vanishing, whereas the mean accelerations do vanish. If example, *P*, is Gaussian then
3.10
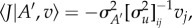
here 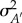 is the organism's acceleration variance (assumed to be isotropic and so like accelerations in the surrounding airstream) and 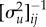
denotes the inverse of the organism's velocity covariance matrix (which, like the airstreams velocity covariance matrix, will be decidedly anisotropic). Equation (3.10) is the three-dimensional analogue of equation (3.5). It is straightforward to show (see electronic supplementary material) that the average magnitude of the jerks is smallest along the mean wind line (or to right of the mean wind line in an Ekman spiral atmosphere in the Northern Hemisphere).

### (c) Further remarks

The postulated effect requires three timescales, two of which are provided by the turbulence: the ‘integral’ timescale (which characterizes the larger, energy-containing motions) and the ‘dissipation’ timescale (which characterizes the smallest motions where turbulence is dissipated as heat). The third timescale (which allows for jerks) is provided by the insect and its aerodynamic response time. It is important to note that although airborne insects would experience jerks, they are a peculiarity of being carried along by a turbulent flow, and will not be evident in measurements made at a fixed location (e.g. by sonic anemometers). Likewise, the effect (like other turbulence cues) would be very difficult to investigate in the laboratory, which is why we advocate an indirect approach when looking for evidence such as a bias in headings of insects apparently attempting to orientate downwind (see above).

If turbulence cues are used to align an insect approximately with the direction of the wind flow, could the jerks be used to distinguish whether it was pointing upwind from pointing downwind? Examination of equation (3.10) reveals that jerks in the downwind direction tend to have an upward component, while those in the upwind direction tend to have a downwind component, so this coupling could, in principle, be used to distinguish the two directions.

Lastly, we speculate that jerks as orientation cues could ‘come for free’ evolutionarily, because being jerked about would amount to a loss of flight control, and so would be naturally avoided; a by-product of this would be downwind flight orientation. If it is indeed the case that migrants do not use visual cues for orientation under certain circumstances, then this mechanism provides a tentative explanation for how high-flying migrants may orient downwind. It also follows that insects would need to keep track of turbulence-induced jerks in time, in order to minimize them. This invokes the importance of a ‘working memory’ that would remember the average jerks sensed over a period of time, in order to elicit the appropriate orientation changes.

## 4. Migration strategies

**Figure 2.**
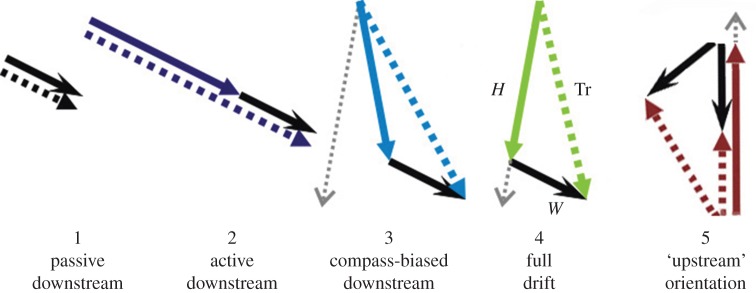
Some orientation responses to wind flow in high-flying migrant insects. Each diagram shows the wind-flow vector (solid black line), the heading vector (solid coloured line, not present in strategy 1 ‘passive downstream movement’) and the resultant track (= displacement) vector (dashed coloured line). The dotted grey line shows the preferred direction of movement (PDM) for those strategies which imply that the insect has one (strategies 3–5 only). Strategy 5 is a variant of ‘full drift’ in which orientation in a seasonally preferred direction has a significant upwind component (in light winds from various directions). Modified from Chapman *et al*. [35].

The various orientation behaviours of both day-flying and nocturnal insect migrants flying within and outside their ‘flight boundary layer’ (the layer of the atmosphere within which the insects' self-powered flight speed exceeds the wind speed, allowing control of migration direction) were detailed in a recent review [43]. Assessing the evidence accumulated about *high-altitude* migration in insects, one can distinguish the following putative strategies (figure 2), which are based on certain of the categories identified by Chapman *et al*. [35] for movement in a fluid medium (air or water).

### (a) Orientation in small insects: quasi-passive downwind transport

This category comprises organisms whose powers of self-propelled locomotion are either non-existent (e.g. aerially dispersing mites or spiders) or very weak (all small insects). Even so, small insects such as aphids can exert some control over when they take off or land, and on their height of flight, so their transport might be termed ‘quasi-passive’ [44]. The airspeeds of the insects involved will make a negligible contribution to their ground speed, and so one might assume that they would show no systematic orientation. This sometimes appears to be the case: radar observations of masses of nocturnally migrating small insects, such as the brown planthopper, *Nilaparvata lugens* (mass approximately 1–2 mg) [45] or rice leaf-roller moth, *Cnaphalocrocis medinalis* (approx. 8 mg) [46], showed no evidence of common orientation, indicating that orientation was random (or perhaps highly multimodal). However, it cannot be assumed that all small migrants' headings will be random because layers of unidentified insects predominantly in the 16–32 mg mass range, flying just after dawn, showed a significant degree of common orientation [47]. Layers of even smaller insects (aphids) flying at dawn also apparently showed orientation patterns [48–50] but the possibility that the pattern could have been due to small numbers of orientating large insects present within the layers was not completely excluded. The environmental cues used to achieve these orientations (which can sometimes be strongly crosswind), and their purpose, remain mysterious but as they are unable to influence the insect's speed and direction significantly, we assume that any adaptive benefit is not directly related to a ‘vector navigation’ strategy.

### (b) Active downwind orientation

The most common wind-related orientation strategy in medium-sized radar-detectable insects is orientation in, or close to, the downwind direction (and here we include nocturnal migrants that show a slight offset due to them being ‘misled’ by the action of the Ekman spiral; see above). As winds at flight altitude are often approximately 8–20 ms^−1^ [3], the option of a track direction significantly different from the downwind direction is severely limited in these insects (with self-powered flight speeds of 1–2.5 ms^−1^), and may well be undesirable as the main benefit of windborne migration is using the power of the wind for long-range movement. Circumstances where simple downwind orientation may be adaptive include:

— migration in arid and semi-arid environments where persistent downwind movement is optimal because it takes insects (e.g. African armyworm moth, *Spodoptera exempta*) towards wind convergence zones where rain is likely to fall [51];
— movement in regions where seasonally prevailing winds happen to take insects in suitable directions (e.g. seasonal movements associated with the Intertropical Convergence Zone (ITCZ) in West Africa [3,51]);
— cases where favourable destination zones can be located in any direction from the source area (for example, nocturnal flight in the eastern spruce budworm moth, *Choristoneura fumiferana*, is consistently oriented downwind [52], probably because suitable (i.e. lightly or undefoliated) stands of host trees do not necessarily lie in any particular direction in the vast boreal forests of eastern North America).

It may be that the intrinsic rates of increase in some species are so high that they can ‘afford’ large migration losses, and a simple distance-maximizing strategy represents the best option.

### (c) Compass-biased downstream orientation

The CBDO strategy, mentioned above, achieves a compromise between moving rapidly and moving in a PDM [35]. The strategy has been well studied in some larger migratory moths in the UK, particularly *A. gamma* [13,16,36,53,54], where it has the following characteristics:

— if the wind on the night in question is highly *unfavourable* for movement in the seasonally preferred direction the migration is suppressed, or limited to short flights only (see also [55]);
— if winds are broadly favourable, but the downstream direction is more than approximately 20° from the PDM, the moths deviate their heading so that it lies *between downstream and the PDM*, but they do not attempt full compensation for drift;
— if winds are *highly* favourable, and the downstream direction is within 20° of the PDM, the migrants do not make significant corrections for drift—in other words, they essentially orient downwind.

High-flying insects generally do not undertake complete compensation, even where this is possible, because the strategy becomes very energy-inefficient as the flow diverges from the PDM, and the migrant makes little progress towards its new habitat. We note that nocturnally migrating songbirds, capable of higher airspeeds than insects, were not always able to fully compensate for drift, even though they often flew at 90° to the wind direction [56], and partial compensation is the most common strategy in nocturnal songbird migrants [54,57–59]. In any case, full compensation is not usually necessary in insects because the migration goal (e.g. an overwintering area) would normally be a broad ecological zone, not a specific location.

Clearly, detailed analysis of this migration strategy depends on being able to identify a PDM, and studying *A. gamma* has the advantage that the species very largely quits the UK (and other parts of Northern Europe) in autumn and re-invades the following spring; so it can be plausibly *assumed* that the PDM is north in spring and south in autumn [15]. Alternatively, the PDM can be estimated from data by a regression method [60], which produces rather similar values (*viz.* 353° in spring and 210° in autumn) [54]. All in all, the migratory orientation of *A. gamma* seems remarkably effective, and the moths' utilization of strong winds blowing in favourable directions allows their ground speeds within a migratory bout to match and sometimes exceed those of songbirds, despite their self-powered airspeeds being only one-third or one-quarter that of songbirds [54,57]. In other cases, interpretation of observed orientation patterns is likely to be much more difficult because, *inter alia*, the place to which the migrant is travelling may well be unknown. Consequently, the observer cannot determine whether the migrant is steering a preordained track in spite of the wind, or whether the wind is drifting the migrant from a track it could have kept to under more favourable circumstances [1, p. 157].

### (d) Full drift

**Figure 3.**
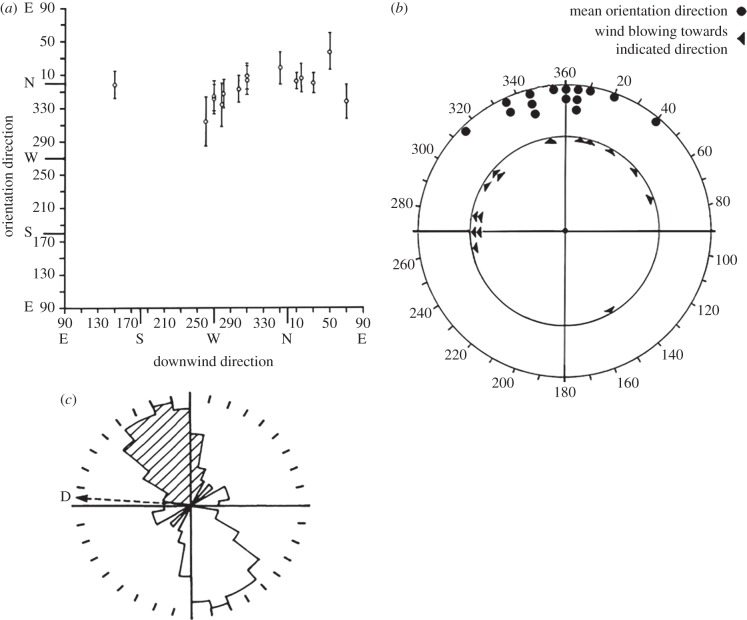
(*a*,*b*) Orientation versus downwind direction for insect targets observed at a radar site at Mara River (1°03′ S, 35°15′ E) in southwestern Kenya, in March 1982. Orientation was approximately towards the north in a variety of downwind directions (west through northeast). (*c*) Example of a crosswind unimodal heading at Mara River 9 March 1982, 20.41–21.02 h, at an altitude range of 540–600 m. The distribution shown is of body *alignment*—an axial quantity, but the shaded section indicates the ‘head end’ direction, deduced from other information. The mean heading was towards 338° (circular s.d. 24.8°), i.e. aligned at 63° to the mean displacement (D) which was towards 270–280° at 11 ± 2 ms^−1^. Wind speed of 10 ms^−1^ was directed *towards* 264°.

In the case of *A. gamma*, described in the last section, orientation is usually fairly close to downwind, but this is not always the case for migrants observed in other situations [4,12]. As the headings become progressively more crosswind, the strategy can approach one of *full drift* where orientation remains approximately constant irrespective of the wind direction (but still with the proviso that oriented groups do not show general backwards drift). An example is provided by orientations of insects at Mara River (1°03′ S, 35°15′ E) in southwestern Kenya (figure 3), which probably included noctuids such as *Spodoptera exempta*. As a whole, the migrants showed a strong propensity to orientate northwards in a range of wind conditions (with downstream directions west through northeast). In some of these cases, the migrants were simply heading downstream (cf. figure 1*a* in [4]), but on other occasions (sometimes on the same night), they were experiencing strong sideways drift (figure 3*c*); for example, their orientation could be approximately 75° from the downwind direction in winds of approximately 10 m s^−1^, when the average insect airspeed was estimated to be 2.5 m s^−1^ [4].

Orientations of this sort can be difficult to interpret. Migratory flight in the strong, easterly winds that usually occurred during the first half of the night in Kenya results in rapid, downwind displacement to the west. In line with this, *S. exempta* outbreaks are known to first spread progressively westwards, but then populations are taken north, eventually into Ethiopia and Yemen [61]. This latter movement is associated with the northward movement of the ITCZ, and a seasonal wind change from northeasterly or easterly to southeasterly or southerly. As seasonally adaptive movements would occur anyway, just due to downwind displacement, how should the strong northwards orientations be construed? Are the migrants attempting to enhance movement in the ‘right’ seasonal direction, or is this over-interpretation of the orientation data in the light of what we know of ‘ultimate’ destinations?

### (e) Orientation towards a fixed directional reference, with upwind displacement

In this case, heading is also in a constant compass direction, but unlike the *S. exempta* moths mentioned in the previous section—who seemed to ignore or be unaware of the severe drift that they were undergoing—migrants only show the present type of orientation when their airspeed is greater than the wind speed. In other words, certain large insects can detect that winds at high altitude are (rather unusually) weak enough for them to move in a fixed geographical direction with a distinct upwind component, and do so move—see the example of the northeastwards-moving grasshoppers in Mali (§1). Here the observations were all made at one site, and this could be a site-specific response, namely, an adaptive movement up the Middle Niger flood plains against the prevailing northeast trade winds [62]. Again, however, some caution is needed in the attribution of adaptiveness to directional orientations—we do not know how long the slow upwind movements were maintained, for example, and there may be other (more mechanistic) explanations. For example, day-flying desert locusts in lighter winds tended to head persistently into wind as a result of an optomotor response (e.g. [25]). Such upwind movements do not result in any notable displacements, however, and *long-range* locust displacements are downwind.

In the wandering glider dragonfly (*Pantala flavescens*) migrating at night over the Bohai Sea in eastern China during late summer, there were occasions when the wind was light, even at altitude, so that the migrants could orientate to the southwest and displace in approximately the same direction, regardless of how the wind direction changed [63]. (This species is, incidentally, known to compensate for wind drift, and to optimize flight speed in response to wind, when flying near the surface [64]). Displacement southwestward in late summer in China is evidently adaptive because it facilitates movement to the latitudes warm enough for the dragonflies to overwinter [63].

In butterflies there is abundant evidence that seasonal migrations take place in PDMs, particularly where movements are largely independent of the wind direction because the migrants are flying near the ground (within their ‘flight boundary layer’) using a solar-based compass [43,64,65]. Therefore, there seems every reason to expect that if conditions were suitable for movement in a fixed geographical direction at high altitudes it would occur; this behaviour is, after all, equivalent to the compass-mediated elements involved in partial compensation strategies (such as CBDO). The mechanism of the compass sense in night-fliers is unknown, but the most likely bases are, perhaps, the Earth's magnetic field [66] or a time-compensated celestial cue (such as the band of the Milky Way) [67,68].

## 5. Synthesis and inter-phylum comparisons

Radar-based investigations of insect orientation at high altitudes have combined case studies of instructive events with (more recently) extensive analyses of large datasets. Some remarkable phenomena have been revealed, and progress made in understanding the proximate (sensory) mechanisms influencing the observed orientations, and how these facilitate migration outcomes in some circumstances. Nonetheless, many uncertainties remain. One of the most problematic is the extent to which the insects use an apparently obvious cue: the visual perception of apparent ground movement. It seems difficult to believe that insects would not take advantage of this mechanism, particularly in conditions of relatively high illuminance (daylight or moonlight), given the superlative motion sensitivity of their visual systems. As mentioned above, however, there are certain night-time situations where a combination of very slow angular rates of background movement and very low reflectance values seem to militate against the use of the visual sense. Additionally, there are crosswind orientation scenarios where the skewness of the observed heading distributions do not accord with predictions of an optomotor-type response.

Considering the competing mechanism—various small-scale anisotropies in turbulent flow that provide cues as to the wind direction—some key predictions of this hypothesis have been met. In particular, our studies found the systematic bias in heading offsets expected when Ekman dynamics were likely to prevail (i.e. in a stably stratified nocturnal boundary layer) but not when such conditions were unlikely (i.e. in convective daytime conditions). It seems, therefore, that nocturnal insect migrants make considerable use of turbulence cues to align themselves with respect to the wind direction. The wind-related orientation mechanism employed by day-flying migrants is still unclear. We note that during the daytime in the UK, surface wind direction is a good predictor of direction at ‘cruising flight’ altitudes (G Hu, SJ Clark, JW Chapman 2016, unpublished data) so, in theory, migrants could detect the wind direction by optomotor means while near the surface, and compare this direction with, say, a time-compensated sun compass cue, and then decide whether or not to abort migration. If the wind direction was favourable they could continue to ascend, maintaining direction with respect to that compass cue and progress in a favourable direction even if they were no longer able to monitor ground image movement.

A more radical suggestion would be that high-flying insects do not make use of visual cues for wind-related orientation because their reactions to atmospheric turbulence, necessary for maintaining flight control, already provide a built-in mechanism for wind-finding (§3c). A more cautious position would be that the high-altitude wind-sensing, like most orientation behaviours, is likely to involve more than one sensory modality, and migrants integrate elements of both visual and mechanosensory reception, with one or other predominant depending on circumstances. We also need to bear in mind that some of the observed orientations (particularly in small insects) may have nothing to do with assisting directional movement.

Finally, we point out some parallels and dissimilarities in turbulence-sensing across various animal taxa in fluid media, as these may not be obvious at first sight. The mechanism proposed by Reynolds *et al*. [21] is a good approximation for smallish insects (with aerodynamic response times less than Lagrangian autocorrelation time of the turbulence) but as the insect size increases it becomes progressively untenable and probably should not be applied to insects with masses > 100 mg (i.e. with aerodynamic response times > 100 ms); it, therefore, would not apply to birds or bats. These size limitations do not apply to the variant of the turbulence theory proposed in Reynolds *et al*. [22], which suggests that larger aerial migrants might be able to use weak turbulent cues to orientate, particularly considering the recent identification of extremely sensitive wind-detecting hairs on bat wings [69]. Considering the new turbulent jerks theory—this would not apply to very small insects (less than or equal to 1 mg), which could not use the orientation cues because they cannot orient (maintain a constant heading) in the presence of turbulence. Large migrants, such as birds and bats, might not be able to detect the cues because their magnitude decreases as the size of the migrant increases, and so the jerks mechanism may not be feasible for these taxa.

Turning to orientation in marine flows, there is evidence that some pelagic animals, including fish [70], jellyfish [71] and juvenile turtles [72], may be able to orientate with respect to ocean currents, e.g. showing positive rheotaxis (facing into the current), where there are no obvious visual, tactile or hydrodynamical cues. The question is: are there any parallels between the putative turbulence mechanisms employed in wind-related orientations and turbulence mechanisms that might provide cues as to water current direction (taking into account that turbulent cues in marine flows will be weaker than in those in the atmosphere). Among the available directional cues are the current shears in the surface Ekman layer of the ocean due to wind stress, or detection of the movements of surface waves themselves. However, there are plenty of other (non-turbulence) possibilities for orientation in relation to the flow, e.g. large-scale odour plumes [73]. As many oceanic gyres are predictable, orientation could be achieved in an indirect way, e.g. by some map sense (using magnetic information [73,74]) and an evolved preferred direction. It seems likely that rheotactic orientation involves multisensory cueing.

## 6. Conclusion

Although there is much that we still do not understand, the identification and evaluation of putative mechanisms for directed responses to flow has recently developed in novel and sometimes surprising ways, as evidenced by the utilization of turbulence cues by high-flying insect migrants. The present addition of a ‘jerks’ model has augmented the robustness of the turbulence-sensing hypothesis. With the benefit of hindsight these mechanisms were hiding in plain view, but their identification nonetheless exemplifies the value of multidisciplinary approaches. In order to make real progress, however, the putative sensory mechanisms for detecting turbulent fluctuations, accelerations and ‘jerks’ need to be identified. Insects are known to detect air flow cues via the antennal Johnston's organs, or cephalic hair system, but it is not clear if the same system could enable the sampling of ‘jerks’. These are open questions, which we hope will be addressed in the next few years. Additionally, broad comparative studies across phyla moving through water and air may provide new insights into the generality of flow-detection mechanisms, as has been achieved by similar approaches for other kinds of movement phenomena [54,75,76].

## Supplementary Material

Supplementary material
