# Supplementary material for: Orientation in high-flying migrant insects in relation to flows: mechanisms and strategies
Source: Philos Trans R Soc Lond B Biol Sci. 2016 Sep 26;371(1704):20150392. doi: 10.1098/rstb.2015.0392 (PMC4992716; doi:10.1098/rstb.2015.0392)
Supplement: Supplementary material [file rstb20150392supp1.pdf]

PHILOSOPHICAL TRANSACTIONS B

Phil. Trans. R. Soc. B.
doi:10.1098/rstb.2015.0392

Supplementary Material for:

Orientation in high-flying migrant insects in relation to flows: mechanisms and strategies

Andy M. Reynolds, Don R. Reynolds, Sanjay P. Sane, Gao Hu and Jason W. Chapman

Correspondence to: Jason Chapman (jason.chapman@rothamsted.ac.uk)

Autoregressive stochastic processes and turbulence modelling

The stochastic equation for turbulent accelerations, Eqn. (2) in the main text, is effectively a 2nd-order autoregressive process. The theory of such processes is well known (Priestly 1981) and is here summarized in terms of turbulence models. As first noted by Sawford (1991) autoregressive processes provide a hierarchy of stochastic models for the position, *x*, velocity, *u* and acceleration, *A*, of a body being carried along a turbulent flow. At the lowest (zeroth) order in this hierarchy, the position of the body is modelled as a Markovian process, at first order the position and the velocity of the body are jointly Markovian, and at second order the position, velocity and acceleration are collectively Markovian. Physically, the hierarchy corresponds to the neglect of memory effects at zeroth order, to the inclusion of a timescale, *T*, representative of the largest (energy-containing) scales of motion, at first-order, and to the inclusion of the Kolmogorov timescale, t_η , representative of the smallest (dissipative) scale of motion, at second-order. The hierarchy can be written as,

$$\begin{aligned} dx &= \sqrt{2K}\,dW \\ du &= -\alpha u dt + \sqrt{2\alpha\sigma_u^2}\,dW \\ dA &= -\alpha_1 A dt - \alpha_2 u dt + \sqrt{2\alpha_1\alpha_2\sigma_u^2}\,dW \end{aligned} \qquad \text{(A1)}$$

where K is the turbulent diffusivity, σ_u^2 is the velocity variance and quantity *dW* is Gaussian noise with mean zero and variance *dt*. Notice that this hierarchy forms the basis for three separate models that are not used jointly. With the diffusion model, *x* is modelled and *u* and *A* are undefined. With the Langevin equation, *u* is modelled directly, x is obtained by integrating u in time, and *A* is undefined. Finally, with the 2nd-order model *A* is model directly and *u* and *x* are obtained by successive integrations in time.

It can be shown (Sawford 1991) that the 2nd-order model is equivalent to a stochastic model for velocity, u,

$$\frac{du}{dt} = -\beta_1 u + f \qquad \text{(A2)}$$

where *f* is a random force (Gaussian distributed with mean zero) that is exponentially correlated with time scale $1/\beta_2$, $\alpha_1 = \beta_1 + \beta_2$ and $\alpha_2 = \beta_1\beta_2$.

Since their introduction by Sawford (1991), second-order regressive models of particle accelerations in turbulent have been shown to be in close agreement with experiment and with data from direct numerical simulations (see e.g., Lamorgese et al. 2007, Reynolds 2003, Reynolds 2005).

Flying to the right of the mean wind line

According to our theory, Eqn. 10, the average size of jerks experienced by a migrant, $\langle J_i | A, v \rangle$, is minimized when the smallest diagonal component of

$$\left[\sigma_u^2\right]_{ij}^{-1} \equiv \frac{1}{\langle uu\rangle\langle vv\rangle - \langle uv\rangle\langle uv\rangle}\begin{bmatrix} \langle vv\rangle & -\langle uv\rangle \\ -\langle uv\rangle & \langle uu\rangle \end{bmatrix} \tag{A3}$$

is minimized, where $\langle uu\rangle$ and $\langle vv\rangle$ are the variances of the migrant's velocity parallel to and orthogonal to its heading, and $\langle uv\rangle$ is the associated covariance. To facilitate analysis $\langle uu\rangle$, $\langle vv\rangle$ and $\langle uv\rangle$ can be approximated by the variances and covariance of the turbulent velocities, u' and v', of the surrounding airstream. These two sets of velocities are related by $u = u'\cos\theta - v'\sin\theta$ and $v = u'\sin\theta + v'\cos\theta$ where θ is the angle between the migrant's heading and the mean wind direction. It follows from this that

$$\begin{aligned} \langle uu\rangle &= \langle u'u'\rangle\cos^2\theta - 2\langle u'v'\rangle\cos\theta\sin\theta + \langle v'v'\rangle\sin^2\theta, \\ \langle vv\rangle &= \langle u'u'\rangle\sin^2\theta + 2\langle u'v'\rangle\cos\theta\sin\theta + \langle v'v'\rangle\cos^2\theta, \\ \langle uv\rangle &= \left(\langle u'u'\rangle - \langle v'v'\rangle\right)\sin 2\theta + 2\langle u'v'\rangle\cos 2\theta \end{aligned} \tag{A4}$$

The largest value of $\langle uu\rangle$ is attained when $\tan 2\theta = \dfrac{-2\langle u'v'\rangle}{\langle u'u'\rangle - \langle v'v'\rangle}$ and in this case $\langle vv\rangle$ is minimized and $\langle uv\rangle$ =0 so that

$$\left[\sigma_u^2\right]_{ij}^{-1} = \begin{bmatrix} \langle uu\rangle^{-1} & 0 \\ 0 & \langle vv\rangle^{-1} \end{bmatrix} \tag{A5}$$

The smallest diagonal component $\left[\sigma_u^2\right]_{ij}^{-1}$ is therefore minimized when the migrant is flying at an angle θ to the mean wind line, i.e., is flying downwind $(\theta = 0)$ when $\langle u'v'\rangle = 0$ and is flying to the right $(\theta > 0)$ of the mean wind line when $\langle u'v'\rangle < 0$; a condition which in the Northern Hemisphere is characteristic of the presence of the Ekman spiral.

References

Lamorgese AG, Pope SB, Yeung PK, Sawford BL. 2007 A conditionally cubic-Gaussian stochastic Lagrangian model for acceleration in isotropic turbulence. *J. Fluid. Mech.* 582, 423–448.

Priestly MB. 1981 *Spectral analysis and time series.* London, Academic Press.

Reynolds AM. 2003 Superstatistical mechanics of tracer-particle motions in turbulence. *Phys. Rev. Lett.* 084503.

Reynolds AM, Mordant N, Crawford AM, Bodenschatz E. 2005 On the distribution of Lagrangian accelerations in turbulent flows. *New J. Phys.* 7, article 58.

Sawford BL. 1991 Reynolds number effects in Lagrangian stochastic models of turbulent dispersion. *Phys. Fluids A* 3, 1577–1586.
